# Executive Function and Mood: The Moderating Role of Athletic Expertise

**DOI:** 10.1177/0031512520987364

**Published:** 2021-01-27

**Authors:** Robert S. Vaughan, Christopher McConville

**Affiliations:** 1 School of Education, Language, and Psychology, York St John University, York, UK; 2 School of Psychology, University of Ulster, Coleraine, UK

**Keywords:** athletic expertise, executive function, mood, positive and negative affect

## Abstract

Executive function (EF) is known to be influenced by mood, but whether this relationship holds for populations of athletes and whether athletic expertise moderates it is uncertain. Thus, in the current study, we examined relationships between positive and negative affect (i.e., mood), the lower-order cortical aspects of executive function (i.e., inhibition, shifting and updating), and athletic expertise. A sample of 256 participants (55.08% male; *M*_age_ = 20.69) completed a self-report mood measure and computerized tests of EF. Individuals with more athletic expertise reported higher positive affect and better EF scores, whereas those with less athletic expertise reported higher negative affect. Structural equation modelling indicated that positive affect was related to better inhibition, shifting, and updating performance but was not related to performance errors. Similarly, negative affect was related to better EF, except for the inhibition latency score. Athletic expertise moderated all significant associations between mood and EF and higher expertise facilitated higher EF performance. Together, athletic expertise is an important individual differences factor in understanding the influence of mood on EF performance.

## Introduction

Interest in executive functioning (EF), the cognitive control processes that regulate thought and behavior (Miyake et al., 2000), has increased significantly in recent years, due to research evidence of an EF influence on all aspects of human performance (e.g., sport; Sakamoto et al., 2018). Executive function has been found to be influenced by mood (i.e., propensity to experience positive or negative emotions; Watson et al., 1988) in the general population. For example, EF performance variability has been found to increase in affective situations (e.g., performance contexts; Gabel & McAuley, 2018). The sports domain may be particularly relevant for examining the link between mood and EF, since elite athletes use a combination of cognitive and affective self-control to make better decisions and perform better (Vaughan et al., 2019). While much research to date has focused on differences in either mood or cognitive control across levels of athletic expertise (Jacobson & Matthaeus, 2014; Lowther & Lane, 2002; Vancini et al., 2019), little prior research has focused on the implications of these findings for the relationship between mood and EF among athletes. Yet, understanding this association between thoughts and feelings is important in sports, given their significant separate contributions to sport performance (Davis & Jowett, 2014; Verburgh et al., 2014). Furthermore, this relationship may differ as a function of athletic expertise (e.g., cognitive performance increases alongside athletic expertise; Vaughan & Edwards, 2020). Thus, in the present study, we examined whether athletic expertise moderates the relationship between mood and EF.

### Mood and Athletes

Affective states (e.g., anger and happiness) are important for sport competition, and they partially explain variations in athletic performance (Davis & Jowett, 2014; Martinent & Nicolas, 2017). Pleasant and unpleasant emotions or moods are experienced on a continuum of low to high intensity, and they are organized into two high-order dimensions known as positive and negative affect (Watson et al., 1988). Typically, positive affect is characterized by pleasurable and optimal states of energy, whereas negative affect is characterized by unpleasurable and distressing states (Watson et al., 1988). As positive and negative affect are considered orthogonal dimensions, individuals may experience high, low, or mixed levels of both. In contrast to emotions, moods tend to be less intense, more enduring, and usually lack a specific reference (Larsen, 2000).

In general, previous investigations indicate that positive affect is associated with advantageous outcomes such as optimal performance, while negative affect has been related to adverse outcomes such as inferior performance (Martinent & Nicolas, 2017). Yet, positive and negative affect may have separate advantages and limitations for athletes (Nicolas et al., 2014). For example, both positive and negative affect can be perceived as either facilitative or debilitative for performance, despite their pleasant or unpleasant valence, depending on the situational context and on individual differences (e.g., athletic expertise; Nicolas et al., 2014; Vancini et al., 2019). In sport, mood variability has a unique relationship with sport performance that may be dependent on the competitive experience (Lowther & Lane, 2002). Furthermore, those with more athletic expertise have tended to interpret their affective state, positive or negative, as more facilitative compared to those with less athletic expertise (Mellalieu et al., 2009).

### Executive Function Among Athletes

Executive functions are important goal-orientated cognitive control processes involved in everyday activities, such as planning and problem-solving (Miyake et al., 2000). Investigators interested in EF in sport have focused on a lower-order model of three interrelated yet distinct cortically mediated, top-down EF components. Specifically, these are (a) *shifting* between tasks, operations, or mental sets (e.g., ensuring that changing demands are adapted to); (b) *inhibition* of dominant responses (e.g., suppression of cognitive and behavioral tendencies caused by internal or external stimuli); and (c) *updating* of working-memory (e.g., monitoring incoming information as well as replacing information that is no longer relevant; Miyake et al., 2000).

To date, high levels of these EF components have been associated with better sport performance, but this influence has varied with individual athlete differences, including athletic expertise (Hagyard et al., 2021). Moreover, assessment of EF across differing levels of athletic expertise has produced contrasting findings. For example, athletes with more athletic expertise have shown better inhibition, shifting, and updating performance, compared to those with less athletic expertise across a range of sport types (Vaughan & Edwards, 2020). Other investigators showed significant differences among athletes with high expertise on some EF tasks (e.g., problem-solving and inhibition) but not others (e.g., decision-making and working-memory) in both team and individual sports (Jacobson & Matthaeus, 2014; Verbugh et al., 2014). Finally, some investigators have reported no significant EF performance differences between high- and low-level athletes (e.g., ice-hockey players) on tasks like inhibition and updating (Lundgren et al., 2016).

Inconsistencies in this literature may be explained by methodological differences between studies, including (a) variations in the tasks that were used to measure EF, (b) failing to capture EF complexity by using only single outcome measures of efficiency or effectiveness, and/or (c) investigators’ use of different taxonomies to portray athlete expertise (Furley & Wood, 2016; Jacobson & Matthaeus, 2014; Swann et al., 2015; Vaughan & Edwards, 2020). Recent, research has shown that EF may interact with other processes to best explain sport performance (Vaughan & Laborde, 2020). Considering the respective importance of both mood and EF for athletes of differing expertise levels, these two factors are likely to interact in their influence on performance.

### Mood and Executive Function

Since moods are less susceptible to regulatory processes and more vulnerable to cognitive disruption than emotions, moods may have a significant independent influence on an individual’s EF (Gabel & McAuley, 2018). Simpson et al. (2014) highlighted a complex link between cognition and mood in non-athlete participant samples for whom there was a significant relationship between spatial working-memory and affect (positive and negative). Positive affect was found to contribute to sustained attention on a pattern matching task, and negative affect was associated with fewer spatial working-memory errors. Cserjési et al. (2009) reported a positive relationship between positive affect and faster response times on the Trails Making Test A and B (Reitan & Wolfson, 1985). They also reported a negative relationship between positive affect and performance on the D2 attention endurance test (Brickenkamp, 1994) such that positive affect was associated with poorer attentional capacities. In a separate study, He and Yin (2016) reported a positive relationship between negative affect and shifting, as indexed by faster performance on the Trails Making Test A and B; but these investigators found no associations between negative affect and EF measures of planning, inhibition and updating, and no relationship between positive affect and any EF tasks.

Gabel and McAuley (2018) reported a positive relationship between negative affect and a latent inhibition measure consisting of a stop-signal, flanker, and spatial-compatibility task (meaning that negative affect was associated with a poorer performance on this measure). They also found a negative relationship between negative affect and a latent working-memory variable consisting of an operation span, reading span, and letter number sequencing task (meaning that this task was performed worse in the context of negative affect). Both effects however were insignificant (i.e., direct effects between EF and positive and negative affect were nonsignificant), except in the presence of emotional reactivity as a moderator, which reversed direction of effects (meaning that better EF performance was associated with higher negative affect). Thus, understanding the affect and EF link may be enhanced with the inclusion of important moderators.

As noted above, the use of single outcome measures in the performance tasks of the foregoing studies has been seen as a significant research design limitation. Investigators are agreed that EF is highly complex, and EF related performance involves processes of both effectiveness (e.g., accuracy and errors) and efficiency (e.g., latency). According to EF theory, differentiating the effects of these processes on performance is important (e.g., see Eysenck et al., 2007, for a review of Attentional Control Theory). For example, Verburgh et al. (2014) reported that athletes with more expertise make more effective, but not necessarily more efficient, decisions. Additionally, much of this work (see He & Yin, 2016, for an exception) has failed to test a model of EF, using instead a selection of different cognitive tasks across a series of separate studies (e.g., Cserjési et al., 2009; Gabel & McAuley, 2018; Simpson et al., 2014). This approach has limited our understanding of the relationship between mood and EF in their effects on sport performance.

Systematic literature reviews have provided a theoretical basis for appreciating the relationship between affect and EF (Gabel & McAuley, 2018; Mitchell & Phillips, 2007). First, cognitive load theory (Sweller et al., 1998) has proposed that affect decreases performance on EF tasks by placing additional demands on cognitive resources that reduce attentional capacity. Second, the mood-as-information theory (Schwarz & Clore, 1983) posits that EF performance differs in the presence of positive and negative affect. Specifically, positive affect is associated with the absence of threat promoting automatic thinking that impedes EF task performance; whereas negative affect is associated with threats promoting analytical thinking that supports EF task performance. Finally, positive affect activates a network of positive cognitions that facilitate cognitive performance on novel or interesting cognitive tasks (e.g., measures of EF). Mitchell and Phillips (2007) reported that, while more work was needed to verify this theory, positive affect is differentially related to EF (e.g., it is positively related with shifting, negatively related to updating, and inconsistently related to inhibition). Negative affect is more variable in its relationship with EF (e.g., studies report significant, nonsignificant, and moderated relationships with working-memory and inhibition). Mitchell and Phillips (2007) recommended that future investigators should investigate potential moderators of the relationship between affect and EF. As both constructs vary across athletic expertise levels (Jacobson & Matthaeus, 2014; Lowther & Lane, 2002; Mellalieu et al., 2009; Swann et al., 2015; Vancini et al., 2019; Vaughan & Edwards, 2020), the relationship between them likely changes as a function of athletic expertise.

### The Present Study

Based on the aforementioned literature and theory, we hypothesized that athletic expertise might moderate the relationship between affect and EF. In a review of 91 studies on athletic expertise, Swann et al. (2015) concluded that a final consensus is difficult to reach because of heterogeneity in the ways past investigators classified athletic expertise. Swann et al. (2015) proposed a standardized taxonomy across sport type accommodating the highest level of performance in terms of success, experience, the competitiveness of the sport, and the global representativeness of the sport; this proposal has received support from others (Hagyard et al., 2021; Vaughan & Laborde, 2020).

It is also difficult to draw on previous work based on the general population of sports participants due to limitations in how investigators have measured EF. In this study, we propose a robust EF examination with reliable tests that can differentiate abilities in the lower-order model of EF (i.e., Cambridge Neuropsychological Test Automated Battery; CANTAB). Additionally, we sought to examine positive and negative affect together in order to provide a more complete and accurate assessment of mood than might be obtained by examining each dimension separately. Our current work had two aims: (i) to assess the relationship between positive and negative affect and EF, and (ii) to determine whether athletic expertise moderated this relationship. We hypothesized that positive and negative affect would be related to inhibition, shifting, and updating, but we specified no direction of effects due to inconsistent prior findings in the literature.

## Method

### Procedure

For this study, we used a quasi-experimental design with purposive sampling. We collected data individually in designated laboratories under test conditions at university sport or psychology departments (e.g., participants were sat down at a desk in a well-lit quiet room without distraction). Participants completed the PANAS, followed by the IED, SST, and SWM in a counterbalanced order, taking all tests on a GIGABYTE 7260HMW BN touchscreen computer running a Pro Windows 8 operating system with a high resolution 13-inch display. Following testing, we thanked and dismissed the participants. We then retrieved and collated data from the CANTAB and entered it into SPSSv25® software for coding, cleaning, and further analyses.

### Participants

A power analysis (assuming power of .80, a medium effect size of .12, and *p* < .05) led to an estimated required sample size of 250 (G*Power; Faul et al., 2007). We recruited a sample of 256 healthy volunteers aged 18–25 years (*M_age_* = 20.69 ± *SD* = 2.03; 55.08% male). Participants were recruited via their sports coaches and tutors, and they were awarded course credit for participation.

We based athletic expertise classifications on Swann et al.’s (2015) taxonomy,^1^ resulting in the following participant subgroups: (a) non-athletes (n = 57), (b) novices (n = 55), (c) amateurs (n = 52), (d) elites (n = 49), and (e) super-elites (n = 43). Athlete participants had competed in a range of external-paced sports such as soccer, hockey, and rugby (Singer, 2000), whereas non-athletes had not participated in any competitive sport (Swann et al., 2015). Before participants began, they read and signed informed consent forms accompanied by information sheets. The study was approved by a university ethics committee in the United Kingdom.

### Materials

**Positive and Negative Affect Schedule (PANAS; Watson et al.,** 1988**).** To measure participants’ affect, we used the PANAS, consisting of two 10-item scales assessing positive (e.g., “interested”, “excited”, and “determined”) and negative (e.g., “afraid”, “distressed”, and “nervous”) affect. On the PANAS, participants rated the degree to which they felt positive or negative on a five-point Likert scale ranging from 1 (“not at all”) to 5 (“extremely”), with higher scores reflecting higher experiences of that affect. Prior research has supported the psychometric properties of the PANAS with athlete and non-athlete populations reporting internal consistency coefficients between α = .83 – .89 and two clear factors via confirmatory factor analysis (Davis & Jowett, 2014; Nicolas et al., 2014; Watson et al., 1988).

**Cambridge Neuropsychological Test Automated Battery (CANTAB®).** To measure the participants’ EF or lower level cortically mediated EF skills, we administered three subtests from the CANTAB (http://www.camcog.com). All investigators hold doctoral qualifications in psychology and were trained in CANTAB administration (i.e., undertook specialised training provided by CANTAB in operating the software, administrating tasks to participants, and analysing EF data). We assessed shifting via the Intra-Extra Dimensional Set Shift Test (IED; Robbins et al., 1994), inhibition through the Stop Signal Task (SST; Robbins et al., 1994), and updating using the Spatial Working-Memory Test (SWM; Robbins et al., 1994). The CANTAB, including these measures, has been reported as a reliable and valid measure of EF in athlete and non-athlete populations with high internal consistency, test-retest coefficients, and modest associations with other established neuropsychological tests of EF (Robbins et al., 1994; Smith et al., 2013; Syvaoja et al., 2015; Vaughan & Edwards, 2020; Vaughan & Laborde, 2020; Vaughan et al., 2019).

The IED measures visual discrimination and shifting. Six geometric shapes in differing colors, appeared on the screen. Participants matched responses with target stimuli and made subsequent decisions based on feedback from the previous trial. If participants chose the correct match, the screen lights up green. Each stimulus represents one dimension (e.g., shape) and then, as participants progress through stages of the test, stimuli next represent two dimensions apiece (e.g., line and shape). Rule changes occur after six or eight correct responses. The task terminated after 50 trials if a participant failed to learn a rule; thus, not all participants completed all stages. Outcome measures were: IED-error (i.e., number of errors made) and IED-stages (i.e., number of stages successfully completed).

The SST assesses response inhibition. Participants were instructed to use a two-button press pad to record their responses to an on-screen arrow stimulus pointing either left or right. The buttons on the press pad corresponded to a direction of the arrow (‘go’ stimulus). In 25% of the trials, an auditory ‘stop’ signal was presented. Participants were instructed to withhold their motor response on presentation of the ‘stop’ signal. Five blocks of 64 test trials were separated by short rest breaks. Outcome measures included: SST-Correct (i.e. the mean reaction time on correct trials), and SST-Stops (i.e. the percentage of correct trials requiring inhibition of the dominant response).

The SWM assesses spatial working-memory and indexes updating. Participants were presented with colored boxes across the screen in a random pattern, and they were instructed to search behind each box for the location of a blue token (i.e., using a process of elimination). Points were awarded for locating tokens. Tokens were hidden behind a different box within the same trial and had to be relocated. Therefore, participants had to recall where the token was previously found and remember *not* to revisit those colored boxes. The color and position of the boxes changed with each trial to prevent the use of a set search strategy. Outcome measures included: SWM-Strategy (i.e., the number of boxes used for each new search with lower scores representing better performance) and SWM-Errors (i.e., participant selected a box where the token had previously been located).

### Data Analysis

We first screened the data for outliers, missing data, and checked for normality of data distribution to ensure all variables met the assumptions of parametric statistical analysis. We extracted descriptive statistics and Cronbach Alpha’s (α) for all necessary variables with a .70 cut-off required for stability (Tabachnick & Fidell, 2007). We used a one-way analysis of variance (ANOVA) to determine differences between athletic expertise groups on all EF measures from CANTAB. This was followed by zero-order correlations to examine relationships between variables.

**Figure 1.**
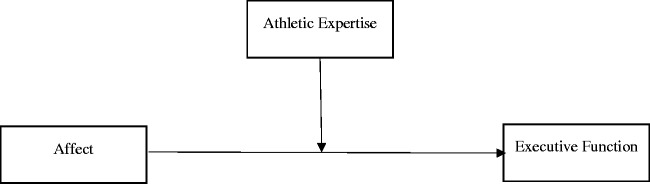
Hypothesized Moderation Model of Athletic Expertise on the Affect and Executive Function Relationship.

We used structural equation modelling with MPlus 7.4 (Muthen & Muthen, 2017) when analyzing EF data in order to examine the relationship between the variables as recommended by Miyake et al. (2000). We used the Comparative Fit Index (CFI), the Tucker Lewis Index (TLI), the standardized root mean square residual (SRMR), and the root mean square error of approximation (RMSEA) to assess goodness of fit using the maximum likelihood with robust standard errors estimation (to control for the categorical nature of the moderator). Following recommendations, values below .08 for the SRMR, below .06 for the RMSEA, and above .90 for the CFI and TLI indicated an acceptable model fit (Hu & Bentler, 1999). Six models were tested - one for each EF outcome measure – in order to avoid issues with multi-collinearity and to ease interpretation with increased interactions (Akinwande et al., 2015). Moderation predictors were mean-centered before interaction terms were calculated. A schematic of our models is available in Figure 1.

## Results

### Preliminary Analyses

Descriptive statistics showing participants’ scores on all variables and internal consistency for the PANAS are displayed in Table 1. Data were screened for multivariate outliers via Mahalanobis distance, and we found no outliers larger than the critical value (χ^2^(3) = 3.34, *p* < .01; Tabachnick & Fidell, 2007). As Box’s M was non-significant (*p* > .05), data were collapsed across gender for subsequent analyses. As age was not significantly correlated with any of the test variables, age was not added as a covariate (*p* > .05). Results of the ANOVA modelling indicated that those with more athletic expertise reported higher positive affect and obtained better EF scores; those with less athletic expertise reported higher negative affect (see Table 1).

**Table 1.** Descriptive Statistics, Internal Consistencies, and Zero-Order Correlations.

	M (SD)								Correlations						
Measure	Total	Super-elite	Elite	Amateur	Novice	Non	ηp^2^	α	1	2	3	4	5	6	7
1.Positive Affect	4.01 (.69)	4.44 (.54)	4.19 (.59)	4.08 (.63)	3.83 (.68)	3.61 (.76)	.05*	.74							
2.Negative Affect	2.08 (.66)	1.62 (.51)	1.89 (.54)	2.11 (.61)	2.28 (.66)	2.49 (.73)	.06*	.78	−.09						
3.IED-Error	15.87 (12.11)	14.02 (12.55)	14.39 (13.52)	15.08 (13.21)	16.09 (12.74)	16.71 (11.29)	.06**	−	−.07	−.16**					
4.IED-Stages	7.02 (.88)	8.81 (.84)	8.09 (.79)	7.91 (.87)	7.42 (.95)	7.07 (.98)	.09**	−	.12*	.15**	−.22**				
5.SST-Correct	487.23 (157.26)	427.36 (61.21)	452.83 (68.45)	478.68 (91.14)	519.95 (99.91)	551.03 (107.63)	.05*	−	−.10*	−.08	.13*	−.25**			
6.SST-Stops	.59 (.17)	.67 (.11)	.61 (.12)	.58 (.14)	.55 (.17)	.51 (.15)	.06*	−	.13*	.11*	−.11*	.26**	−16**		
7.SWM-Strategy	20.97 (6.52)	14.64 (4.36)	16.61 (4.43)	20.02 (6.12)	23.29 (6.53)	26.13 (5.74)	.11**	−	−.15*	−.17**	.14*	−.22**	.19**	−.25**	
8.SWM-Error	22.53 (15.82)	18.78 (9.74)	20.15 (10.41)	22.29 (12.06)	23.96 (14.12)	24.88 (15.01)	.10**	−	−.05	−.14*	.24**	−.21**	.20**	−.24**	.19**

Note. N = 256. IED = Intra-extra dimensional shift, SST = Stop Signal Tasks, SWM = Spatial Working-Memory.

* p < .05. ** p < .01.

### Structural Equation Modelling

Main effects were tested before adding interaction terms, as MPlus provides limited information of model fit for moderation analyses (Maslowsky et al., 2015). We tested six models for each EF outcome, using positive and negative affect as predictors. Results indicated acceptable fit (RMSEA = .045 – .057; SRMR = .055-.067; CFI = .901 – .939; TLI = .909 – .947); therefore, we proceeded by adding interaction terms (see Table 2). Again, the model fit was acceptable across all models and, in most cases, the model fit demonstrated moderate improvements explaining 14–28% (*R*^2^ = .14–.28) of the variance between EF with athletic expertise and mood (i.e., positive and negative affect).

**Table 2.** Standardised Main and Interaction Effects of Positive and Negative Affect and Athletic Expertise on Executive Function.

	IED-Error			IED-Stages			SST-Correct			SST-Stops			SWM-Strategy			SWM-Error		
	Δ*R*^2^	β	SE	Δ*R*^2^	β	SE	Δ*R*^2^	β	SE	Δ*R*^2^	β	SE	Δ*R*^2^	β	SE	Δ*R*^2^	β	SE
Predictors	.18**			.23**			.14**			.21**			.28**			.19**		
Positive Affect		−.02	.05		.10**	.06		−.08*	.05		.10**	.04		−.13**	.03		−.02	.06
Negative Affect		−.12**	.04		.12**	.05		.03	.06		.09*	.05		−.14**	.03		−.12**	.05
Athletic Expertise		−13**	.05		.14**	.04		−.10*	.06		.14**	.04		−.14**	.04		−.13**	.05
Positive Affect × Expertise		−.05	.06		.13**	.04		−.12**	.05		.15**	04		−.22**	.03		.07	.05
Negative Affect × Expertise		−.18**	.04		.15**	.03		.07	.05		.12**	.05		−.25**	.03		−.20**	.03
Model fit indices																		
RMSEA		.051			.045			.054			.046			.042			.049	
SRMR		.062			.055			.064			.057			.053			.059	
CFI		.911			.934			.905			.926			.945			.918	
TLI		.919			.944			.914			.937			.953			.929	

Note. N = 256. IED = Intra-Extra Dimensional Set Shift, SST = Stop Signal Task, SWM = Spatial Working-Memory.

*p < .05. **p < .01.

Athletic expertise yielded a positive association with all EF measures, specifically with higher expertise related to greater shifting (i.e., fewer IED-Error and more IED-Stages), greater inhibition (i.e., more SST-Stops and shorter SST-Correct latencies), and greater updating (i.e., fewer SWM-Error and lower SWM-Strategy).

Higher positive affect was associated with better shifting (i.e., more IED-Stages), better inhibition (i.e., greater SST-Stops and shorter SST-Correct latencies), and better updating (i.e., lower SWM-Strategy). Nonetheless, positive affect was unrelated to IED-Error and SWM-Error. The positive affect x expertise interaction followed a similar pattern, in that, higher positive affect and higher expertise was associated with better shifting performance (i.e., more IED-Stages), better inhibitory performance (i.e., more SST-Stops and faster SST-Correct latencies), and better updating (i.e., lower SWM-Strategy). The inclusion of athletic expertise as an interaction (i.e., positive affect x expertise) did not change effects pertaining to errors (i.e., IED-Error and SWM-Error) and these remained non-significant.

Higher negative affect was associated with better shifting (i.e., less IED-Error and more IED-Stages), better inhibition (i.e., more SST-Stops), and better updating (i.e., fewer SWM-Error and fewer SWM-Strategy), but higher negative affect was unrelated to SST-Correct (i.e., reaction time on trials requiring withdrawal of the dominant response). The inclusion of athletic expertise (i.e., negative affect x expertise) showed comparable results, such that a combination of higher negative affect and higher expertise was related to better shifting (i.e., fewer IED-Error and more IED-Stages), better inhibition (i.e., more SST-Stops), and better updating (i.e., fewer SWM-Error and fewer SWM-Strategy). Interestingly, the inclusion of the negative affect x expertise interaction did not change the effect for SST-Correct and it remained non-significant.

## Discussion

We aimed first to assess the relationship between affect and EF and, second, to determine whether athletic expertise moderated this relationship. In line with previous research, participants with more athletic expertise performed better on tasks of inhibition, shifting, and updating; and they reported higher positive affect. Also consistent with past findings, participants with less athletic expertise reported higher negative affect (Jacobson & Matthaeus, 2014; Lowther & Lane, 2002; Mellalieu et al., 2009; Swann et al., 2015; Vancini et al., 2019; Vaughan & Edwards, 2020; Verburgh et al., 2014). Our results also supported our predictions in that positive affect was related to greater accuracy and lower latencies but not errors, while negative affect was related to greater accuracy, higher latencies, and fewer errors on measures of inhibition, shifting and updating. In addition, athletic expertise positively moderated affect and EF relationships.

The current study represents a methodological advance that may account for differences between our own and previous findings. First, our use of Swann et al.’s (2015) categorization of athletic expertise may have provided greater precision in detecting effects. That is the inconsistencies in definition and dichotomous measurement of athletic expertise may have distorted effects in previous studies. Second, our use of a range of lower level but reliable outcome measures (Smith et al., 2013; Syvaoja et al., 2015) from an established EF model (Miyake et al., 2000) enabled us to better capture EF complexity which, despite other investigators’ recommendations, have not been commonplace in prior literature. According to Attentional Control Theory (Eysenck et al., 2007), EF performance involves effectiveness (e.g., accuracy and errors) and efficiency (e.g., latency) across both goal-directed and stimulus-driven systems, and these may be differentially affected by emotional dispositions (Coombes et al., 2009). Although hypothetical, this account may transfer to the sport context, offering possible explanations for findings such as those of Verburgh et al. (2014), who reported that athletes with more expertise make more effective, but not necessarily more efficient decisions, and indeed the current findings.

With some methodological improvements over previous research in the way that we categorized athletic expertise and measured EF, our data provided partial support among athletes for prior findings of a link between mood and EF among the general population. In line with Simpson et al. (2014) we found that negative affect was associated with fewer spatial working-memory errors. Simpson et al. (2014) did not measure inhibition or updating, reducing our ability to make direct comparisons to their study. Our finding of a positive association between positive affect and shifting was also reported by Cserjési et al. (2009) who found a positive relationship between positive affect and faster reaction times on the Trails Making Test A and B. He and Yin (2016) also reported a positive relationship between negative affect and shifting performance, but, in contrast to our findings, He and Yin (2016) reported no effects between measures of planning, inhibition and updating with negative affect and no relationship between positive affect and any EF measure. It is likely that these differences across studies could be explained by variation in sampling which might have affected EF (e.g., age; Miyake et al., 2000). Our participants were healthy volunteers aged 18-25 years, whereas Simpson et al. (2014) recruited elderly participants, Cserjési et al. (2009) recruited females with an obesity diagnosis, and He and Yin (2016) recruited families with children.

There may be neurochemical links between affect and EF that are enhanced for athletes (Mitchell & Phillips, 2007). For example, affect may impact neurotransmitter synthesis from increased exercise/physical activity, affecting behavior in turn (e.g., executive function task performance; Young, 2007). Brain imaging research supports this idea, as both affect and EF demonstrate an inverted U-shape relationship with physical activity (Stroth et al., 2010). That is, physical activity is associated with increases in EF and affect until an optimum level and then EF and affect decrease after this point. Physical activity is likely to increase to optimum levels with increases in athletic expertise. Research also supports a cognitive stimulation hypothesis whereby increased cardiovascular fitness mediates the relationship between physical activity and EF (Tomporowski et al., 2008). Huijgen et al. (2015) investigated the interactive effect of training hours on EF in elite and sub-elite youth soccer players. Huijgen et al. (2015) found that elite players showed better inhibitory performance on SST testing than their sub-elite counterparts. Moreover, Mitchell and Phillips (2007) noted that affect and EF activate similar areas of the prefrontal cortex, making it likely that they share some neurological basis.

Following recommendations from Mitchell and Phillips (2007), our research is the first to have examined the moderating effect of athletic expertise on the mood and EF relationship. Gabel and McAuley (2018) highlighted the importance of individual differences for explaining the link between affect and EF. These investigators reported no relationship between positive and negative affect with indices of inhibition and working-memory. However, when emotional reactivity was added as a moderator, the negative affect and EF relationship became significant (i.e., negative with inhibition [reaction time] and positive with working-memory). Likewise, we found that athletic expertise moderated the affect and EF relationship. While the inclusion of athletic expertise as a moderator did not change the relationship between affect and EF (e.g., significance or direction), it did augment the direct effects reported. For example, higher positive affect was associated with more SST-Stops and shorter SST-Correct latencies, and higher negative affect was associated with lower SWM-Strategy and SWM-Errors (i.e., indicative of better updating performance), with this effect increasing across the athletic expertise continuum (i.e., greater effects for those with more expertise). It is possible that those with more athletic expertise better regulate their affective states to maintain EF performance (e.g., as experience of elite level sports competition increases; Vaughan et al., 2019). While somewhat speculative, athletes with more expertise may be more experienced in dealing with intense affective states, regardless of valence (Lowther & Lane, 2002; Mellalieu et al., 2009; Nicolas et al., 2014; Vancini et al., 2019). That is, maintaining EF performance while in a negative mood, such as those experienced when sport performance is below expectations may be essential to being successful, and may be akin to learning how to negotiate different intensities of neural activation, thus placing less demand on the prefrontal cortex and reducing demands on attentional capacities (Johnson, 2001). This hypothetical account warrants further research testing.

There may be some notable theoretical explanations for our findings. Cognitive load theory indicates that affective states overload attentional capacities and therefore reduce EF performance. In contrast, we found that both positive and negative affect were related to better EF performance, providing little support for this theory. Jacobson and Matthaeus (2014) reported that participation in sport may result in cognitive skills transfer (i.e., transfer of sport-specific cognitive skills into the general cognitive domain), that may, in turn, increase attentional capacity. Our findings partially support the mood-as-information theory positing that positive and negative affect impacts EF differently, such that positive affect promotes automatic thinking that hinders performance and negative affect promotes analytical thinking that improves EF performance. We observed that negative affect was negatively related to shifting and updating errors, suggesting a more analytical approach, whereas positive affect was unrelated to errors. Similarly, positive affect was related to faster inhibition reaction times, suggesting a more automatic approach, whereas negative affect was unrelated to reaction time. Theory also indicates that positive affect activates positive cognitions facilitating EF task performance (Mitchell & Phillips, 2007). While our findings support this notion, we analyzed positive and negative affect concurrently as orthogonal dimensions (Watson et al., 1988), finding that both were related to better EF performance. Previous investigators may have examined positive and negative affect in isolation or presumed that a high score in one assumes the absence of the other. Our findings indicate that in the sport context, higher scores in both positive and negative affect, activate cognitive networks that facilitate EF performance.

### Implications

There are some important theoretical and practical implications of these findings. First, investigators could consider affect as a potential antecedent in Attentional Control Theory (Eysenck et al., 2007). It is possible that affective states predispose an individual’s likelihood to engage either the (more efficient) goal-directed or stimulus-driven (resource demanding) systems. Activation of the stimulus-driven system over the goal-directed system (i.e., when the central executive becomes overloaded and EF performance decreases), has negative implications for performance which may be potentially exacerbated or offset by specific affect × EF interactions. Second, research indicates that EF training (e.g., working-memory via the adaptive n-back paradigm) has significant benefits for athletes’ attentional control under pressure conditions (Ducrocq et al., 2017). Thus, intervention work aimed at training EF should consider athletes affective state at pre- and post-EF assessment, particularly when considering effectiveness and efficiency outcomes.

### Limitations and Future Directions

While strengths of this study include a newly accepted framework for categorizing athletic expertise and reliable EF indices, our study had limitations that warrant discussion. For example, we relied upon self-report measures of affect that may be subject to response biases such as social desirability. Also, recent investigations have made improvements to the PANAS by including a direction scale, capturing a four-factor model of intensity and direction for both positive and negative affect (Nicolas et al., 2014). Recently investigators have adopted more specific conceptualizations of affect to determine their relationships to EF. For example, Shields et al. (2016) found that two aspects of negative affect, namely anxiety, but not anger, impaired EF performance on a card sorting task. It is plausible that anxiety, an avoidance-motivated emotion, and anger, an approach-motivated emotion, engage different motivational systems with different effects on EF (Carver, 2006). Review work attests to the importance of differentiating approach-avoidance motivations for athlete performance (Lochbaum & Gottardy, 2015). Thus, future work should further deconstruct positive and negative affect to further investigate these trends. Finally, our cross-sectional design limited our ability to draw conclusions regarding causality and direction of influence between these variables. Future work should replicate the current research with longitudinal designs that enable an examination of consistency and changing effects over time (e.g., a competition season).

## Conclusions

In this article, we detailed the rationale, method, and results of the first examination of the relationship between mood and EF in sport. With regard to the EF components of inhibition, shifting, and updating, we found that positive affect was related to greater accuracy and lower latencies but not errors, while negative affect was related to greater accuracy, higher latencies, and fewer errors. In addition, athletic expertise positively moderated these relationships. These findings extend our understanding of these constructs by differentiating the outcomes of EF tasks and highlighting a more complex association between variables. We emphasized the need for and specified the nature of future research with athletes in this domain.

## Note

1 Athletic expertise was calculated as: [(A + B + C/2)/3] × [(D + E)/2], where A was the athlete’s highest level of performance, B was success at the athlete’s highest level, C was experience (in years) at the athlete’s highest level, D was competitiveness of sport in athlete’s country, and E was global representativeness of sport. Athletes were tagged as novice (a score between 1–4), amateur (a score between 5–8), elite (a score between 9–12) super-elite (a score between 13–16). Those who failed to score on Swann and colleagues’ (2015) criteria were tagged as non-athletes (a score of 0). We used the tags, non-athlete – super-elite in line with previous work (e.g., Vaughan & Edwards, 2020; Vaughan & Laborde, 2020).
